# Distributed cerebellar plasticity implements adaptable gain control in a manipulation task: a closed-loop robotic simulation

**DOI:** 10.3389/fncir.2013.00159

**Published:** 2013-10-09

**Authors:** Jesús A. Garrido, Niceto R. Luque, Egidio D'Angelo, Eduardo Ros

**Affiliations:** ^1^Neurophysiology Unit, Department of Brain and Behavioral Sciences, University of PaviaPavia, Italy; ^2^A. Volta Physics Department, Consorzio Interuniversitario per le Scienze Fisiche della Materia, University of Pavia Research UnitPavia, Italy; ^3^Department of Computer Architecture and Technology, University of GranadaGranada, Spain; ^4^Brain Connectivity Center, C. Mondino National Neurological InstitutePavia, Italy

**Keywords:** cerebellar nuclei, long-term synaptic plasticity, gain control, learning consolidation, modeling

## Abstract

Adaptable gain regulation is at the core of the forward controller operation performed by the cerebro-cerebellar loops and it allows the intensity of motor acts to be finely tuned in a predictive manner. In order to learn and store information about body-object dynamics and to generate an internal model of movement, the cerebellum is thought to employ long-term synaptic plasticity. LTD at the PF-PC synapse has classically been assumed to subserve this function (Marr, 1969). However, this plasticity alone cannot account for the broad dynamic ranges and time scales of cerebellar adaptation. We therefore tested the role of plasticity distributed over multiple synaptic sites (Hansel et al., 2001; Gao et al., 2012) by generating an analog cerebellar model embedded into a control loop connected to a robotic simulator. The robot used a three-joint arm and performed repetitive fast manipulations with different masses along an 8-shape trajectory. In accordance with biological evidence, the cerebellum model was endowed with both LTD and LTP at the PF-PC, MF-DCN and PC-DCN synapses. This resulted in a network scheme whose effectiveness was extended considerably compared to one including just PF-PC synaptic plasticity. Indeed, the system including distributed plasticity reliably *self-adapted* to manipulate different masses and to learn the arm-object dynamics over a time course that included fast learning and consolidation, along the lines of what has been observed in behavioral tests. In particular, PF-PC plasticity operated as a *time correlator* between the actual input state and the system error, while MF-DCN and PC-DCN plasticity played a key role in generating the *gain controller*. This model suggests that distributed synaptic plasticity allows generation of the complex learning properties of the cerebellum. The incorporation of further plasticity mechanisms and of spiking signal processing will allow this concept to be extended in a more realistic computational scenario.

## Introduction

The cerebellum plays a critical role in the precise control of movements, as is evident when studying patients with cerebellar malfunctioning and diseases (Thach, 1996). The cerebellum receives proprioceptive signals (Sawtell, 2010) and *copies* of motor commands (Schweighofer et al., 1998a) together with haptic information (Ebner and Pasalar, 2008; Shadmehr and Krakauer, 2008; Weiss and Flanders, 2011) through MFs. By means of these signals and its own internal circuitry, the cerebellum is able to learn and process sensorimotor information, and thereby regulate the initiation, intensity and duration of motor acts in an anticipatory manner (Spencer et al., 2005; Manto et al., 2012). This *gain control* operation is a fundamental aspect of motor control in animals, as it allows not only the rapid regulation of motor acts according to contextual cues, but also, through learning, adaptation of these acts to bodily and environmental changes. This *adaptable gain control* requires *closed-loop* interactions between command centers and effectors and is thought to involve the cerebellum embedded in the so-called *forward controller* loop (Schweighofer et al., 1998a; Wolpert et al., 1998; Wolpert and Ghahramani, 2000). In fact, the abstraction of models (kinematics and dynamics) of objects under manipulation (Shadmehr and Mussa-Ivaldi, 2012) is efficiently achieved thanks to close interaction between the cerebral and the cerebellar cortex (Middleton and Strick, 2000; Wang et al., 2008). However, two main issues remained unresolved. First, the adaptable gain controller localized in the cerebellum is thought to require suitable learning and memory mechanisms, whose nature is still debated. Secondly, it remains to be explained how a gain control system involving the cerebellum is able to optimize its performance in the face of broad and varying operative ranges.

Several attempts have been made to understand how the cerebellum implements adaptable gain control. The original theories, based on analysis of network connectivity (Marr, 1969; Albus, 1971; Fujita, 1982), defined the cerebellum as a timing and learning machine. The granular layer was hypothesized to perform expansion recoding of input signals and the PF-PC synapse to learn and store relevant patterns under the control of the teaching signal provided by CFs. On the basis of electrophysiological determinations, it has been suggested that the inferior olive (IO), by comparing proprioceptive and predicted signals, is indeed able to provide quantitative error estimation (Bazzigaluppi et al., 2012; De Gruijl et al., 2012). Moreover, some authors, on the basis of eye-movement analysis, have advanced the hypothesis of a two-state learning mechanism (Shadmehr and Brashers-Krug, 1997; Shadmehr and Holcomb, 1997), wherein a fast learning process takes place in the cerebellar cortex (granular and molecular layer, possibly involving PF-PC plasticity) and a slow consolidation process takes place in deeper structures (possibly the DCN) (Shadmehr and Brashers-Krug, 1997; Shadmehr and Holcomb, 1997; Medina and Mauk, 2000). Clearly, in the development of an adequate model of adaptable cerebellar gain control, it has to be known where and how learning actually occurs. Long-term synaptic plasticity is thought to provide the biological basis for learning and memory in neuronal circuits (Bliss and Collingridge, 1993) and appears in various forms of potentiation (LTP) and depression (LTD). In the cerebellum, long-term synaptic plasticity was initially thought to occur only as LTD or LTP (Marr, 1969; Albus, 1971) at the PF-PC synapse, but now synaptic plasticity is known to be distributed and to occur also in the granular layer, molecular layer and DCN (Hansel et al., 2001; Gao et al., 2012). In particular:

1. Synaptic plasticity in the granular layer is unsupervised and may serve to improve spatiotemporal recoding of MF input patterns into new GC discharges [expansion recoding (D'Angelo and De Zeeuw, 2009)].
2. Synaptic plasticity in the molecular layer is supervised and may serve to store correlated granular layer patterns under the teaching signal generated by CFs. This plasticity is in fact composed of multiple mechanisms: PF-PC LTD may occur together with PF-MLI LTP, globally reducing PC responses, while PF-PC LTP may occur together with PF-MLI LTD and MLI-PC LTD, globally increasing PC responses (Gao et al., 2012).
3. Synaptic plasticity in the DCN is supervised and may serve to store correlated granular layer patterns under the teaching signal generated by PCs (Hansel et al., 2001; Boyden et al., 2004; Gao et al., 2012). This plasticity is, in turn, composed of several mechanisms generating MF-DCN (Bagnall and du Lac, 2006; Pugh and Raman, 2006) and PC-DCN (Morishita and Sastry, 1996; Aizenman et al., 1998; Ouardouz and Sastry, 2000) LTP and LTD. On the one hand, it has been suggested that MF-DCN and PF-DCN plasticity are important in controlling cerebellar learning in the context of EBCC (Medina and Mauk, 1999, 2000) and that equivalent forms of plasticity in the VN are important in controlling cerebellar learning in the VOR (Masuda and Amari, 2008). On the other hand, it has been proposed that the nature of cerebellar cortical and nuclear plasticity and the involvement of extra-cerebellar plasticity sites are highly dependent on the task to be performed, e.g., EBCC or VOR (De Zeeuw and Yeo, 2005; Porrill and Dean, 2007; Lepora et al., 2010). In the present context, with the aim of developing a general computational scheme, we have not considered the potential task-dependence of the learning process.

We explored the impact of distributed cerebellar synaptic plasticity on gain adaptation using a robotic control task in a closed loop, starting from the assumption that there are three learning sites, one in the cerebellar cortex (PF-PC) and two in the DCN (MF-DCN and PC-DCN), all generating LTP and LTD. We found that simultaneous recalibration of weights at these multiple synaptic sites was required to implement self-adaptable gain control over a broad dynamic range involving manipulation of objects with different masses. Moreover, the model implied, due to the definition of the learning rules and the configuration of the learning parameters, that learning was faster in the molecular layer than in DCN, supporting adaptation mechanisms on different time scales. This result suggests that distributed synaptic plasticity is needed to generate the complex computational and learning properties of the cerebellum and to improve motor learning and control.

## Methods

A cerebellar model was constructed taking into account the major functional hypotheses concerning the granular layer, the PC layer and the DCN. The main synaptic connections between these structures (PF-PC, PC-DCN, and MF-DCN) were endowed with long-term synaptic plasticity mechanisms. The cerebellar model was embedded into a control loop designed to operate a simulated robotic arm manipulating different masses. The simulator of the robotic arm and the control loop were implemented in *Simulink* (Matlab R2011a), in accordance with previous models (Luque et al., 2011a,b,c; Tolu et al., 2013) (see Appendix B). The cerebellar model was implemented in C++ and was embedded in *Simulink* as an *S-function* block. The source code is available at: https://senselab.med.yale.edu/modeldb/ShowModel.asp?model=150067.

### Cerebellar model

**Figure 1:**
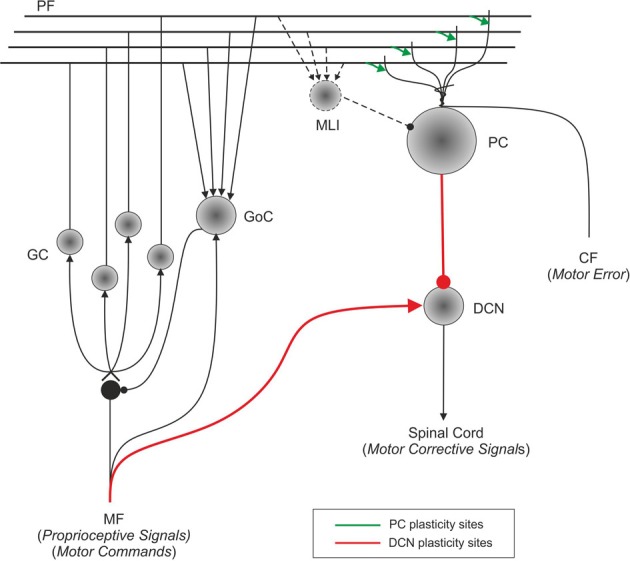
**Schematic representation of the main cell types in the cerebellum and of their connections.** Suggestions about the nature of inputs signals are indicated [according to Schweighofer et al. (1998b)]. The pathways involved in long-term synaptic plasticity are drawn in green (for DCN afferents) and blue (for PC afferents). PF, parallel fiber; MF, mossy fiber; CF, climbing fiber; GC, granule cell; GoC, Golgi cell; PC, Purkinje cell; DCN, deep cerebellar nuclei; IO, inferior olive; MLI, molecular layer interneuron.

**Figure 2:**
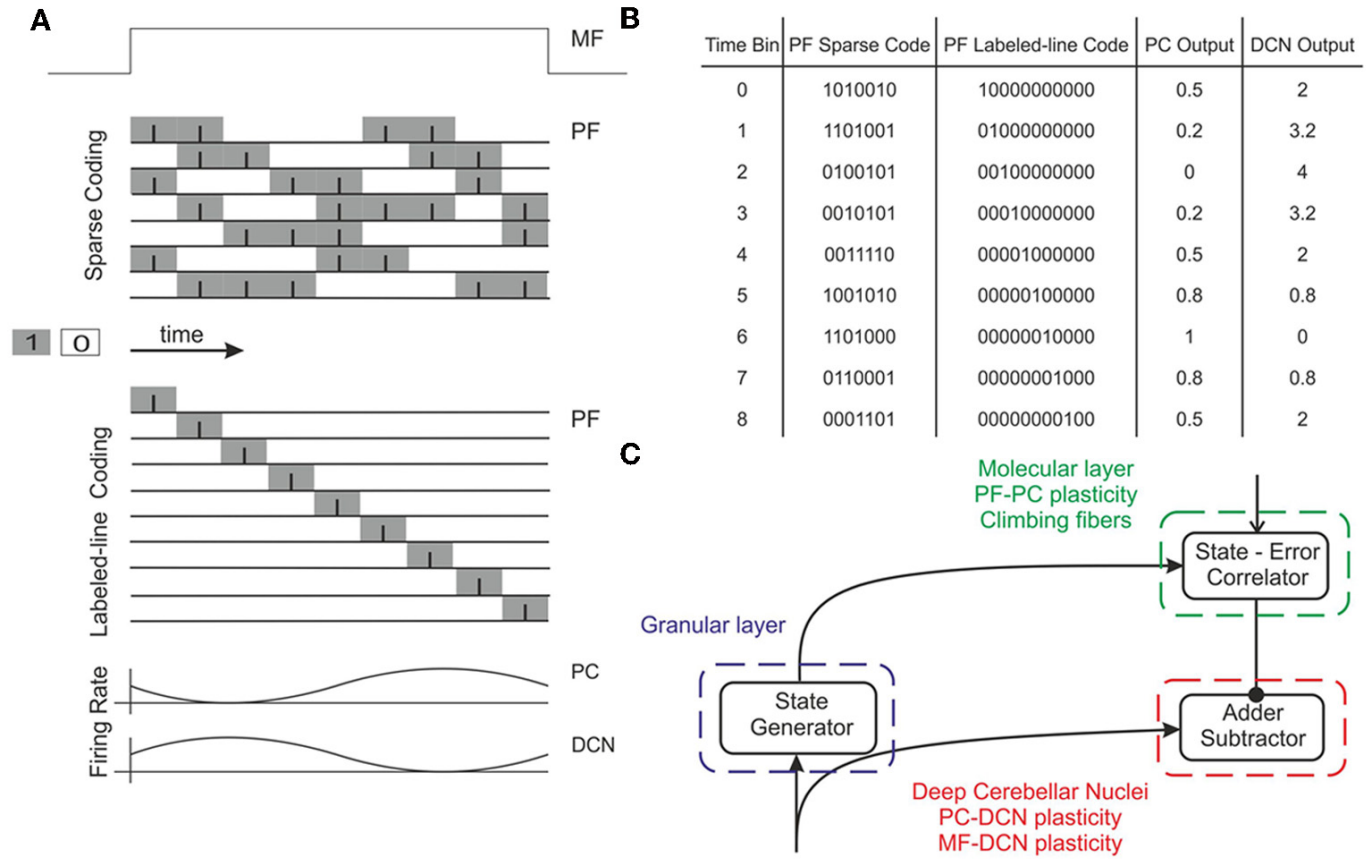
**Working hypothesis of cerebellar learning in a manipulation task.** In our model, the system is further simplified by computing states without explicit spike representation. **(A)** During each manipulation trial, the onset of the movement initiates a *non-recurrent sequence of firing states* in the PFs (Yamazaki and Tanaka, 2007) due to the incoming activity in MFs. Each state is correlated with the error signal, representing the difference between the desired and actual positions of the robotic joint, and reaches the PCs through the CFs. This correlation is thought to occur through plasticity at the PF-PC synapses (Marr, 1969). After repeated pairing of *PF* states and CF error signals, an association is formed between the two; a learned corrective torque occurs and precedes the wrong movement. This association involves either reduction or increase of PC firing rate at different times. Finally, the temporally correlated signals from PCs are inverted (due to the inhibitory nature of the PC-DCN connection) and rescaled before reaching the motor neurons. The figure presents two alternative coding strategies: in our model, there are no spikes and the states correspond directly to the offset from movement onset indicated by the time bin. Formally, this corresponds to passing from a *sparse coding* to a *labeled-line coding*. **(B)** Binary representation of activity in a PF subset (1: active synapses, 0: inactive synapses) and firing rates in the corresponding PC and DCN neuron (in PCs the values are normalized in the range 0–1). A low PC firing rate corresponds to a high DCN firing rate. **(C)** Block diagram of the elements involved in the model. A *state generator* (which is reinitialized with the onset of a new trial) mimics the functionality of the cerebellar granular layer. A *state-error correlator* emulates the PC function: PF-PC long-term plasticity under supervision of CFs. Finally, an *adder/subtractor* receives the inputs coming from the MFs (multiplied by the MF-DCN synaptic weights) and subtracts the signal coming from the PCs (multiplied by the PC-DCN synaptic weights).

The model provides a simplified representation of signal processing, while accounting for the main computational and learning properties of the cerebellar circuit. Each layer of the cerebellum was implemented as a set of parameter values corresponding to the firing rate of the neural population. Consequently, and since the interaction between neuronal layers in the model is linear, “synaptic strength” and “synaptic weight” correspond to gain factors describing the influence that firing frequency in the presynaptic cell group has on the postsynaptic cell group. Thus, like gain, “synaptic weights” are adimensional. An overview of the cerebellar circuit is shown in Figure 1 and of computational features of the model is shown in Figure 2.

#### Signal coding in the cerebellar model

Previous models of cerebellar control of eyelid conditioning assumed that MFs convey spike sequences with a constant firing rate during presentation of the conditioned stimulus (Medina and Mauk, 1999; Yamazaki and Tanaka, 2007, 2009). Accordingly, in the present model, MF activity was represented by a constant firing rate. The MFs received constant signals (1) during the execution of each learning trial, and their input was set to 0 after the trial. It was assumed that, owing to internal dynamics, the granular layer circuit is capable of generating time-evolving states even in the presence of a constant MF input (Fujita, 1982). The CFs were assumed to transmit an error signal (0–1) representing the normalized difference between the desired and actual positions and velocities of each arm joint.

The onset of MF activity started the generation of the granular layer state sequence (see below) and also provided the excitatory drive to DCN cells (Figure 2). The DCN generated the cerebellar output by emitting positive (or zero) corrective torques that were added (with a positive or negative sign depending on whether it corresponded to agonist or antagonist muscles) to the crude inverse dynamic signal coming from the motor cortex.

#### The granular layer

The granular layer was implemented as a state generator (Yamazaki and Tanaka, 2005). When MF activity reaches the granular layer, it produces non-recurrent time patterns that are repeated exactly in each learning trial (Figure 2A). Thus, the relative time offset along the arm plant trajectory is represented by the correlative activation of 500 different states, mimicking the behavior of 500 PFs sequentially activated during movement execution. It should be noted that the procedure adopted here formally corresponds to a *labeled-line* coding scheme (Figures 2A,B).

#### The purkinje layer

The PC layer has been suggested to correlate the PF input activity with the CF error-based teaching signal (Marr, 1969; Albus, 1971). Taking advantage of the state representation occurring in PFs, the PC layer was implemented by means of a look-up table, which associates each actual state with an output firing rate progressively learned along the trial (Figure 2B; see also below the synaptic plasticity section for a comprehensive description of mechanisms). The activity of the PC layer is defined as follows:

(1) $${\text{Pur}}_{i}(t)=f_{i}\left(PF(t)\right),i\in 1,\;2,\ldots \text{ Number of muscles}$$

where Pur_*i*_(*t*) represents the firing rate of the PCs associated with the *i-*th muscle and *f*_*i*_ associates each granular layer state (i.e., one active *PF*) with a particular output firing rate at the *i-*th PC (Figure 2B). In the present 3-joint arm, there are six PCs accounting for the three pairs of agonist-antagonist muscles (one pair per joint).

#### DCN cells

The DCN cells integrate the excitatory activity coming from MFs and the inhibitory activity coming from PCs (Figure 2C). By linearly approximating the influence of excitatory and inhibitory synapses on DCN firing rate, the output of the DCN cell population was described as follows:

(2) $$\begin{array}{l} {\text{DCN}}_{i}(t)=W_{MF-{\text{DCN}}_{i}}-{\text{Pur}}_{i}(t)\cdot W_{PC_{i}-DCN_{i}},\\ \;i\in 1,2,\ldots ,\text{Number of muscles} \end{array}$$

where DCN_*i*_(*t*) represents the average firing rate of the DCN cell associated with the *i*th muscle, *W*_*MF* − *DCN*__*i*_ is the synaptic strength of the MF-DCN connection at the *i*th muscle, and *W*_*PC*_*i*_ − *DCN*__*i*_ is the synaptic strength of the PC-DCN connections at the *i*th muscle. Thus, the DCN layer was implemented as an *adder/subtractor* and the afferent activity coming from the MFs and PCs was scaled by synaptic strengths (MF-DCN and PC-DCN synapses, respectively). These synaptic weights were progressively adapted during the learning process, following the synaptic plasticity mechanisms explained below. It is important to note the absence of an MF activity term. As previously explained, we assume a constant input rate from MFs during the learning process. Thus, the excitatory component of the DCN firing rate is dependent only on the MF-DCN synaptic weight.

### Synaptic plasticity

The cerebellar model included plasticity mechanisms at three different sites: the PF-PC, PC-DCN, and MF-DCN synapses. As a whole, this set of learning rules led the cerebellum toward a relatively fast adaptation using PF-PC plasticity and a subsequent slow adaptation using MF-DCN and PC-DCN plasticity. This allowed the PF-PC synaptic weights to be kept within their optimum functional range through feedback coming from the actual movement. Importantly, the inclusion of the proposed learning rules allowed the cerebellar model to learn, independently, the timing (in the PF-PC synapses) and gain (in the MF-DCN and PC-DCN synapses) of the task.

#### PF-PC synaptic plasticity

**Figure 3:**
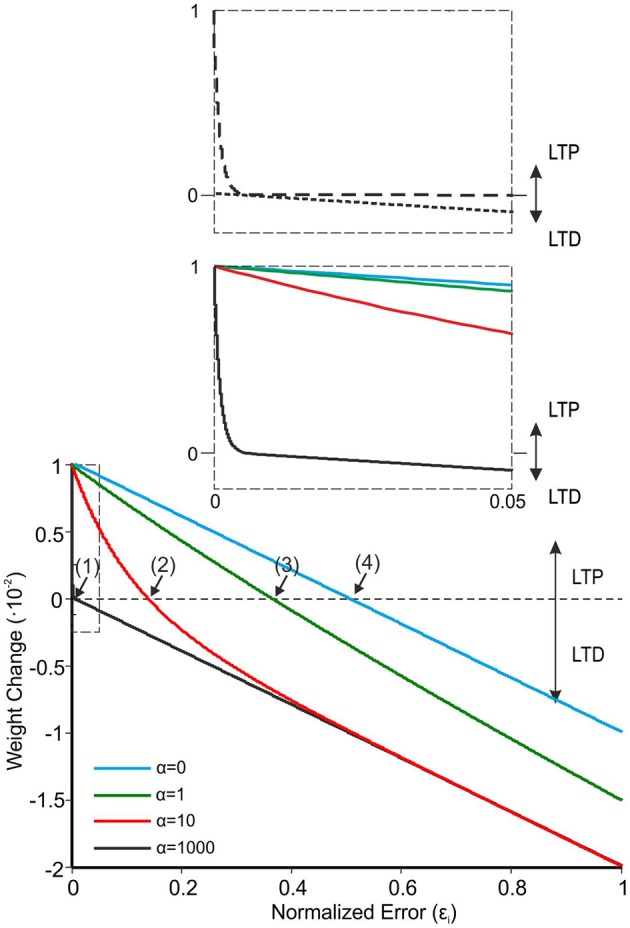
**The learning rule for PF-PC plasticity.** Comparison of four different α values in Equation 3 (LTP_Max_ and LTD_Max_ were set at 0.01 and 0.02). Equation 3, which represents the synaptic weight change as a function of normalized error reaching the cerebellum through the CFs, shows better learning performances at high α values (black solid line). With α = 1000, Equation 3 crosses the *X*-axis at a very low value (curve 1: ε_*i*_ ≈ 4.7·10^−3^). When α is lowered, the curves cross the *X*-axis at progressively higher ε_*i*_ values (curve 2: α = 100, ε_*i*_ ≈ 137.7·10^−3^; curve 3: α = 1, ε_*i*_ ≈ 366·10^−3^; curve 4: α = 0, ε_*i*_ ≈ 500·10^−3^). The inset shows that with α = 1000 there is a rapid decrease of LTP toward zero, while the LTD evolves linearly with the error.

This is the most widely investigated cerebellar plasticity mechanism and different studies have supported the existence of multiple forms of LTD (Ito and Kano, 1982; Boyden et al., 2004; Coesmans et al., 2004) and LTP (Hansel et al., 2001; Ito, 2001; Boyden et al., 2004; Coesmans et al., 2004). PF-PC plasticity was recently observed in alert animals (Márquez-Ruiz and Cheron, 2012). The main form of LTD is heterosynaptically driven by CF activity, and is therefore related to the complex spikes generated by CFs, while the main form of LTP is related to the simple spikes generated by PFs. The present model implements PF-PC synaptic plasticity as follows:

(3) $$\Delta W_{PF_{j}-PC_{i}}(t)=\{\begin{array}{ll} \frac{{\text{LTP}}_{\text{Max}}}{{\left(\varepsilon_{i}(t)+1\right)}^{\alpha }}-{\text{LTD}}_{\text{Max}}\cdot \varepsilon_{i}(t) & \text{if }PF_{j}\text{ is active at }t,i\in 1,2,\ldots ,\text{ Num. of muscles}\\ 0 & \text{otherwise} \end{array}$$

where Δ*W*_*PF*_*j*_ − *PC*_*i*_(*t*)_ is the weight change between the *j*th PF and the target PC associated with the *i*th muscle, ε_*i*_ is the current activity coming from the associated CF (which represents the normalized error along the executed arm plant movement), LTP_Max_ and LTD_Max_ are the maximum LTP/LTD values, and α is the LTP decaying factor. It should be noted that in previous cases when a synaptic weight had to be modified according to a teaching signal, a linear function was used (Masuda and Amari, 2008). However, this implied that while LTD was generated proportionally to the incoming error signal through CFs, LTP was constantly generated when spikes reached the target PC. In this way, plasticity was not able to fully remove the manipulation task error since LTD was always counterbalanced by “unsupervised” LTP. In order to avoid this problem, LTP_Max_ and LTD_Max_ were set to 0.01 and 0.02 and α was set at 1000. This led to a marked decrease of LTP (evolving with the change in ε) and prevented plasticity saturation (e.g., see Figure 3).

In accordance with the assumption that the granular layer operates as a state generator (Yamazaki and Tanaka, 2007), this synaptic plasticity rule modified the strength only of the active PFs. The synaptic weight variation was positive (LTP) when CF activity approached 0 (low error levels in the movement). Otherwise the weight variation was negative (LTD) and was linearly proportional to CF activity.

#### MF-DCN synaptic plasticity

MF-DCN synaptic plasticity, which has been reported to depend on the intensity of DCN cell excitation (Racine et al., 1986; Medina and Mauk, 1999; Pugh and Raman, 2006; Zhang and Linden, 2006), was implemented as:

(4) $$\begin{array}{l} \Delta W_{MF-{\text{DCN}}_{i}}(t)=\frac{{\text{LTP}}_{\text{Max}}}{{\left({\text{Pur}}_{i}(t)+1\right)}^{\alpha }}-LTD_{\text{Max}}\cdot {\text{Pur}}_{i}(t),\\ \;i\in 1,\ldots ,\text{Number of muscles} \end{array}$$

where Δ*W*_*MF* − *DCN*__*i*_(*t*) represents the weight change between the active MF and the target DCN associated with the *i*th muscle, Pur(*t*) is the current activity coming from the associated PCs, LTP_Max_, and LTD_Max_ are the maximum LTP/LTD values, and α is the LTP decaying factor. In order to maintain the stability of the learning process, the LTP_Max_ and LTD_Max_ values had to be lower than those defined at the PF-PC synapse and were set at 10^−3^ and 10^−4^, respectively. As in Equation 3, α was set at 1000, thus allowing a fast decrease of LTP and preventing early plasticity saturation (e.g., see Figure 3).

The MF-DCN learning rule, although formally similar to the PF-PC learning rule, bore two relevant differences. The first is due to the reduced ability of MFs, compared with PFs, to generate sequences of non-recurrent states (Yamazaki and Tanaka, 2007, 2009; Yamazaki and Nagao, 2012). The learning rule in Equation 4 would lead synaptic weights to their local maximum values (one activity value per different state) allowing plasticity to store temporally correlated information. In order to simplify the interpretation of the results, we used a single MF activity state, which was then associated by plasticity mechanisms with different gain values at MF-DCN synapses. The second difference concerns the connection driving LTD and LTP. While PF-PC plasticity was driven by CF activity, MF-DCN plasticity was driven by PC activity. This mechanism can optimize the activity range in the whole inhibitory pathway comprising MF-PF-PC-DCN connections: high PC activity causes MF-DCN LTD, while low PC activity causes MF-DCN LTP. This mechanism implements an effective cerebellar gain controller, which adapts its output activity to minimize the amount of inhibition generated in the MF-PF-PC-DCN inhibitory loop.

#### PC-DCN synaptic plasticity

PC-DCN synaptic plasticity was reported to depend on the intensity of DCN cell and PC excitation (Morishita and Sastry, 1996; Aizenman et al., 1998; Ouardouz and Sastry, 2000; Masuda and Amari, 2008) and was implemented as:

(5) $$\begin{array}{l} \Delta W_{PC_{i}-{\text{DCN}}_{i}}(t)=\frac{{\text{LTP}}_{\text{Max}}\cdot {\text{Pur}}_{i}{\left(t\right)}^{\alpha }}{{\left(DCN_{i}(t)+1\right)}^{\alpha }}-{\text{LTD}}_{\text{Max}}\cdot (1-{\text{Pur}}_{i}(t)),\\ \;i\in 1,\ldots ,\text{Number of muscles} \end{array}$$

where Δ*W*_*PC*_*i*_−*DCN*__*i*_(*t*) is the synaptic weight adjustment at the PC-DCN connection reaching the DCN cell associated with the *i*th muscle. LTP_Max_ and LTD_Max_ are the maximum LTP/LTD values that this learning rule can apply at any time (as with the MF-DCN learning rule, these values were set at 10^−3^ and 10^−4^ respectively), Pur_*i*_(*t*) is the current activity coming from the associated PC (in the range [0,1]), DCN_*i*_(*t*) is the current DCN output of the target DCN cell, and α represents the decaying factor of the LTP (again, it was set at 1000 as in MF-DCN and PF-PC learning rules). This learning rule led the PC-DCN synapses into a synaptic weight range appropriate to match the synaptic weight range at PFs. Equation 5 caused LTP only when both the PCs and their target DCN cell were simultaneously active.

### Control loop and input-output organization

The brain can plan and learn the optimal trajectory of a movement in intrinsic coordinates (Houk et al., 1996; Nakano et al., 1999; Todorov, 2004; Hwang and Shadmehr, 2005). This operation consists of three major tasks: computation of the desired trajectory in external coordinates, translation of the task space into body coordinates, and generation of the motor command (Uno et al., 1989). In order to deal with dynamic variations, the system needs to incorporate a feedback error learning scheme (Kawato et al., 1987) in conjunction with a crude inverse dynamic model of the arm plant.

**Figure 4:**
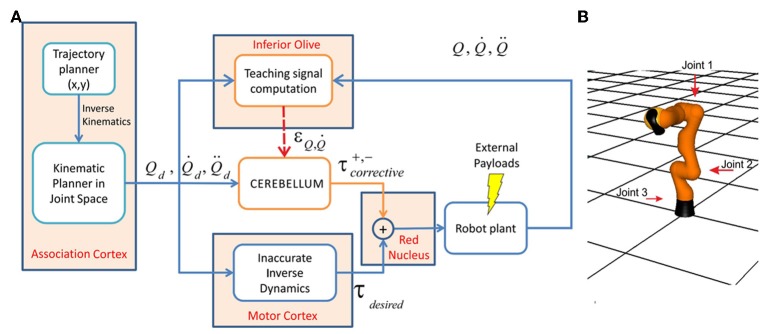
**Control scheme and robotic arm. (A)** Essential control loop used for simulated manipulation tasks. The association cortex generates the desired trajectory (in terms of position, velocity, and acceleration) in body coordinates and the corresponding command signal is transmitted to both the motor cortex and to the cerebellum through the MFs. In the motor cortex command torques are calculated using an inverse dynamic arm model. The cerebellum generates corrective torques compensating for deviations from target trajectory (error) caused by the dynamic interaction of the arm with the object during manipulation. The signals from the motor cortex and cerebellum are added together in the red nucleus and then the output is delivered to the robot arm. The cerebellar corrective torques can be adapted in order to minimize the motor error. This requires a teaching signal generated by the IO. In the IO, the actual state is compared with the desired state in order to obtain the teaching error-dependent signal which reaches the cerebellum through the CFs. **(B)** The LWR arm. The three joints used in our experiments are indicated (red arrows); all the other joints were fixed (made rigid).

It was recently reported that multiple closed loops characterize the input-output organization of cerebro-cerebellar networks (Bostan et al., 2013). It has been proposed that the association cortices provide the motor cortex with the desired trajectory in body coordinates (Figure 4A). In the motor cortex, the motor command is calculated using an inverse dynamic arm model (for a review see Siciliano and Khatib, 2008). The *spinocerebellum-magnocellular red nucleus* system provides an accurate model of musculoskeletal dynamics, which are learned with practice by sensing motor command consequences in terms of executed movements (proprioception). The *cerebrocerebellum-parvocellular red nucleus system*, which projects back to the motor cortex, provides a crude inverse-dynamic model of the musculoskeletal system, which is acquired while monitoring the desired trajectory (Kawato et al., 1987). The crude inverse-dynamic model works together with the dynamic model, thus updating motor commands according to predictable errors occurring when executing a movement. In our control system, only the dynamic model involving cerebellar feedback to actual movement was implemented.

On the basis of these theories, we implemented a control loop using a forward architecture (see Figure 4A), in which only information about sensorial consequences of non-accurate commands was available (i.e., the difference between actual and desired arm plant joint positions). The natural error signal for learning was obtained as the difference between the actual movement and the motor command. This implies that if *M* muscles control a motor system endowed with *N* sensors, the *N* sensory errors must be converted into *M* motor errors (*MxN* complexity). How to use this sensory information to drive motor learning is the so-called *distal error problem* or *motor error problem* (Porrill et al., 2004; Haith and Vijayakumar, 2007). In order to circumvent this problem, the present cerebellar model used the adaptation mechanisms described above, which correlated the actual and desired states toward the generation of an accurate corrective motor command.

The system controller comprised different modules in accordance with studies indicating that the brain first plans the optimal trajectory in task-space coordinates, translates these into intrinsic-body coordinates, and finally generates the appropriate motor commands to achieve these transitions (Houk et al., 1996; Nakano et al., 1999; Todorov, 2004; Hwang and Shadmehr, 2005; Izawa et al., 2012). The system controller was composed of some pre-defined non-adaptive modules and a cerebellar model adapting over the learning trials (Figure 4A). The pre-defined modules, which maintained fixed parameters throughout the trials, independently of the load under manipulation, were the following:

- *Association cortex*. This module operated as a trajectory planner delivering desired positions and velocities of the target trajectory and it included an inverse kinematic model translating this trajectory from Cartesian into arm-joint coordinates.
- *Motor cortex*. This module, based on a recursive Newton-Euler algorithm (RNEA), generated crude step-by-step motor commands implementing the desired trajectory through an inverse dynamic model. The corresponding torque values could drive the robot arm along the desired trajectory in the absence of any external load, but failed to do so when loads were added during manipulation.
- *Red nucleus*. This module added the motor commands provided by motor cortex module to the corrective torques coming from the adaptive cerebellar module.

The cerebellar model is the only adaptive module in the system controller. This module learnt to correct the inverse dynamic model, pre-calculated for the desired trajectory in the absence of external load, in order to manipulate the actual load. The inclusion of three different learning rules allowed the cerebellar model to store the temporal properties of corrective torques in the PF-PC synapses and the gain of corrective torques in the MF-DCN and PC-DCN synapses.

The system integrated a lightweight robot (LWR) simulator within a feedforward control loop (Albu-Schäffer et al., 2011). The physical characteristics of the simulated robot plant were dynamically modified to match different contexts (e.g., the payload to be handled, which translated into a variation of the arm+object dynamics model). The LWR is a 7-degrees of freedom (7-DOF) arm composed of revolute joints. In our experiments, for simplicity, we only used the first, second and fifth joints, while the other joints were kept fixed (Figure 4B). The robot's dynamics were taken into account as indicated in appendix B.

### Manipulation task and experimental protocol: training trajectory

Several reports in the literature have provided evidence of the role played by the cerebellum in complex manipulation-like tasks: (i) animal studies have shown that rapid target-reaching movements (Kitazawa et al., 1998) and circular manual tracking (Roitman et al., 2009) induced error encoding by PCs, (ii) imaging techniques have shown increased cerebellar activation in response to errors occurring during the execution of various tasks including tracking (Imamizu et al., 2000; Diedrichsen et al., 2005), and (iii) more specifically, prediction error has been shown to drive motor learning in saccades (Wallman and Fuchs, 1998) and reaching (Tseng et al., 2007). Thus, PCs are able to produce corrective signals in response to error signals (assumed to reach PCs through the CFs). The proposed model offers an explanation, based on evidence from complex learning tasks but also on theories proposed in relation to EBCC and VOR experiments, of how gain control (required for VOR and manipulation tasks) and timing control (also required for EBCC tasks) might occur in a plausible cerebellar model.

**Figure 5:**
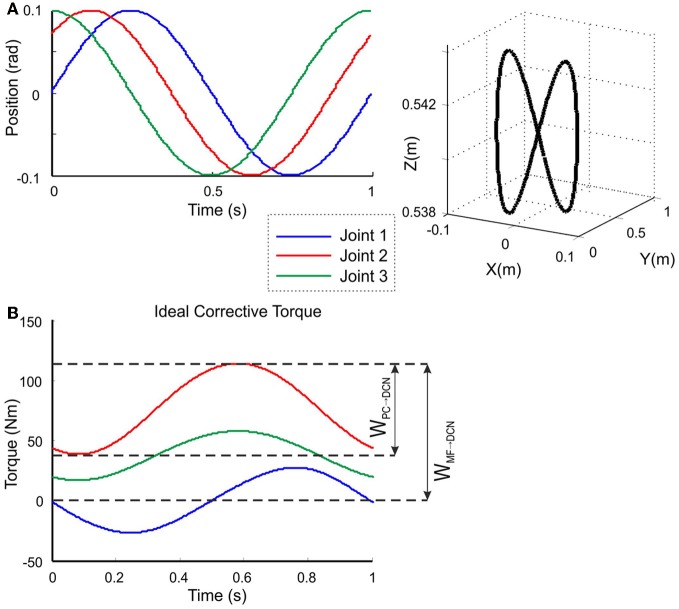
**Calculation of the target trajectory.** Three-joint periodic trajectory defining an 8-shape movement [redrawn with permission from Luque et al. (2011c)] **(A)** Angular coordinates of each joint of the LWR *(left)*, and 3D view of the robot end-effector trajectory in Cartesian coordinates *(right)*. This 8-shape trajectory demands a movement difficult enough to allow robot arm dynamics to be revealed in fast movements (Hoffmann et al., 2007). **(B)** Ideal corrective signals that the cerebellar model had to infer for each of the three joints in order to correct the produced error when manipulating a 10-kg payload. According to the proposed hypothesis, the MF-DCN synaptic weights (*W*_*MF* − *DCN*_) had to adapt to the gain of the maximum torque value at every joint, while the PC-DCN weight (*W*_*PC* − *DCN*_) had to set the maximum inhibition (or torque value subtraction) needed.

The model was tested in a smooth pursuit task (Luque et al., 2011a,b,c), in which the LWR targeted a repeated trajectory using its three revolute joints (Figure 4B). The benchmark *8-shape* trajectory (Figure 5A) was composed of vertical and horizontal sinusoidal components, whose equations in angular coordinates are given for each joint by:

(6) $$q_{1}(t)=A_{1}\cdot \sin(\pi t)+C_{1}$$

(7) $$q_{2}(t)=A_{2}\cdot \sin(\pi t)+C_{2}$$

(8) $$q_{3}(t)=A_{3}\cdot \sin(\pi t)+C_{3}$$

where *A*_*i*_ and *C*_*i*_ are the amplitude and phase of the trajectories followed by each robot joint. The movement for the whole trajectory took just one second with masses requiring considerable corrective torques. This task was chosen to be sufficiently challenging to allow proper assessment of the learning capability of the cerebellar model. The corrective action driven by the cerebellum is especially relevant with respect to inertial components, Coriolis force and friction generated by movement (Schweighofer et al., 1998a). Changing the payload made it possible to assess the dynamics model abstraction capability of the cerebellum. As an example, Figure 5B shows the corrective torque values that the cerebellum should infer when manipulating a 10-kg payload. This corrective torque is calculated for each mass by means of the RNEA, which is able to solve the inverse dynamics problem.

In order to quantitatively evaluate movement performance, the mean absolute error (MAE) of each robot joint was calculated. This performance estimator was monitored in each trial and allowed evaluation of movement accuracy and of its improvement during the learning process.

## Results

As a first step in simulating the 8-shape task, the corrective torques needed for smooth manipulation of different masses (0.5, 1.5, 2.5, 6, and 10 kg) were calculated (Figure 5B). The maximum and minimum torque values for each joint and mass (see Table A1 in Appendix A) were used to estimate the ideal weight values at DCN afferents. It was assumed that, as a consequence of learning, the maximum torque values corresponded to the MF-DCN synaptic weights, while the difference between the maximum and minimum torque values corresponded to PC-DCN synaptic weights. It should be noted that the PC-DCN synapse, by forming the only inhibitory pathway to the cerebellar nuclei, provides the only mechanism capable of reducing the output torques in the model.

### Network activity and motor performance with fixed weights at DCN synapses

**Figure 6:**
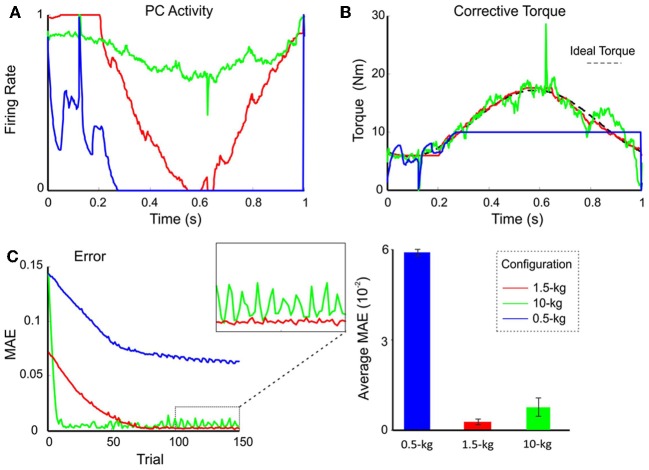
**Performance and learning with different weight configurations.** Plasticity occurred only at PF-PC synapses, and was disabled at the MF-DCN and PC-DCN synapses. The synaptic weights were set at values appropriate for the manipulation of 0.5-kg (*blue lines*), 1.5-kg (*red lines*), and 10-kg (*green lines*) masses. In all three cases, 1.5-kg masses were actually manipulated. **(A)** Normalized activity of the PCs associated with the 2nd joint after 149 learning trials. For clarity, only the behavior of the second joint is shown, but similar results were found along the learning process also in joints 1 and 3. Note that by using the proper weight configuration *(red line)*, PC activity effectively ranged from 0 to 1. It should be noted that the time course of the PC firing rate corresponds to the synaptic weights at the PF-PC synapses (see Methods for explanation). **(B)** Corrective torque values provided by the DCN associated with the 2nd joint after 149 learning trials. **(C)** Evolution of the MAE during the learning process *(left)*. The box highlights the different stability of motor control during the last 50 trials. The histogram *(right)* shows the average MAE calculated over the last 50 trials for different payloads, revealing that smallest MAE values and variability occurred with the proper setting.

In order to evaluate the impact, on the cerebellar circuit, of weights at synapses afferent to DCN, the PC firing rate was monitored after setting the MF-DCN and PC-DCN weights at their ideal values pre-calculated to handle different masses. The PF-PC weights were then allowed to change along a learning process composed of 1-s trial trajectories repeated 150 times. Figure 6A shows the normalized firing rate of one PC during a 1-s trial. The PC firing range changed clearly depending on the payload. It should be noted that in this configuration learning occurred only at the PF-PC synapse. As explained in the Methods, the change in PF-PC synaptic weights corresponds linearly to the change in PC firing rate.

Using the pre-calculated synaptic weight setting for a 1.5-kg payload allowed the PCs to operate over the whole range of firing rates producing, as a consequence, a fine adjustment of the DCN firing rate. This allowed the circuit to approach the ideal theoretical values of PC and DCN activity (Figure 6B) thus optimizing the learning corrective action in terms of stability and accuracy (Figure 6C). However, when DCN afferents were set at values pre-calculated for the manipulation of a heavier mass (10 kg), the PC activity was limited to a small frequency range in order to counteract the gain overscaling at DCN afferent synapses. Likewise, when DCN afferents were set at values pre-calculated for the manipulation of a lighter mass (0.5 kg), the learning process constrained PC activity to saturate to its minimum (no inhibition at DCN cells) along the trial (Figure 6A). These effects reduced the cerebellar output precision (Figure 6B) and made the corrective action unstable, decreasing the learning performance (Figure 6C). These experiments showed that synaptic weights at MF-DCN and PC-DCN connections were crucial to allow the cerebellar model to generate accurate and stable corrective motor outputs when manipulating different masses.

### Network activity and motor performance with adaptable weights at DCN synapses

**Figure 7:**
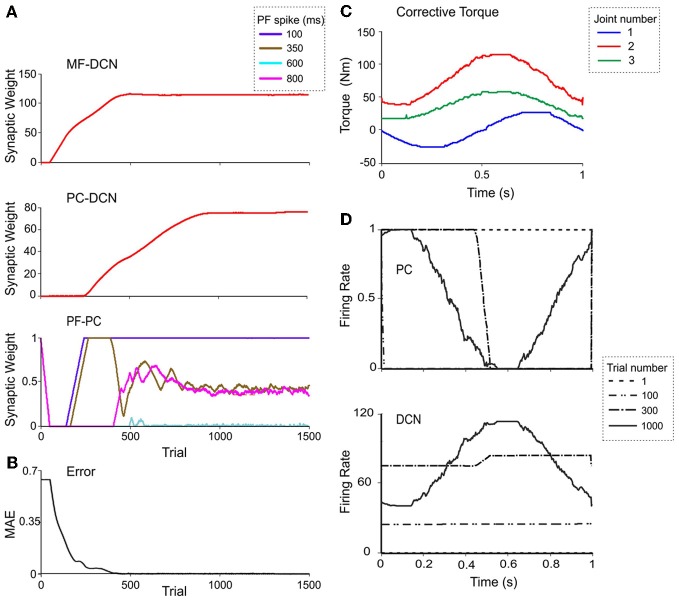
**Weight evolution in the cerebellar model with multiple plasticity mechanisms.** Simulations were carried out using all the plasticity mechanisms (PF-PC, MF-DCN, and PC-DCN) for manipulation of a 10-kg external payload during 1500 trials. Initial synaptic weights allowed accurate movement of the arm without manipulation of any object (0-kg external payload). **(A)** Evolution of synaptic weights at MF-DCN *(top)*, PC-DCN *(middle)* and PF-PC *(bottom)* connections related to joint 2 *agonist* muscle. In PF-PC synapses, four different PFs that become active 100 ms *(purple)*, 350 ms *(brown)*, 600 ms *(cyan)*, and 800 ms *(pink)* after movement initiation are shown. **(B)** Corrective torques along a 1-s movement, provided by the cerebellar model at the 1500th trial in the learning process. Note the similarity of these values with the ideal ones calculated in Figure 5C. **(C)** Evolution of the average MAE of the three joints during the learning process. **(D)** Normalized PC firing rate *(top)* and DCN firing rate *(bottom)* during trials taken at different stages of the learning process: trial 1 *(red)*, trial 100 *(yellow)*, trial 300 *(gray)*, and trial 1000 *(green)*.

In order to investigate the effectiveness of learning rules regulating DCN synaptic weights, a simulation involving manipulation of a 10-kg payload was performed (Figure 7). The synaptic weights of MF-DCN and PC-DCN connections were allowed to self-adjust along a learning process composed of 1-s-trial trajectories repeated 1500 times.

Remarkably, the MF-DCN and PC-DCN synaptic weights tended to stabilize more slowly than those of the PF-PC synapse (Figure 7A) for two main reasons. First, the LTD_Max_ and LTP_Max_ parameters were higher in the PF-PC (10^−2^ and 2·10^−2^ in Equation 3) than in MF-DCN and PC-DCN plasticity mechanisms (10^−3^ and 10^−4^ in Equations 4, 5). These LTD_Max_ and LTP_Max_ values were needed in order to stabilize the learning rules. Second, learning at the MF-DCN and PC-DCN synapses depended on PC normalized activity. Thus, the MF-DCN and PC-DCN synaptic weights changed only when some PF-PC weights tended to saturate (toward 0 and 1, respectively; see above) (Figure 7A). Indeed, the evolution of weights was significantly slower at the PC-DCN than at the MF-DCN synapses. As exemplified for the agonist of joint 2, the MF-DCN weights stabilized in about 800 trials while the PC-DCN weights stabilized in more than 10,000 trials (for a comprehensive list of evolution of weights at the MF-DCN and PC-DCN synapses with different masses, see Table A2 in Appendix A). This slow evolution was caused by the dependence of PC-DCN learning on DCN activity, which in turn depended on MF-DCN and PC-DCN adaptation (detailed information about the PC-DCN synaptic weights after the learning process is shown in Table A3 in Appendix A). In parallel to the evolution of MF-DCN and PC-DCN synaptic weights, PF-PC weights evolved to stable values that were reached after 800 trials (Figure 7A).

After the DCN synaptic weight adaptation process, the cerebellum was able to provide corrective torques pretty similar to those theoretically calculated to solve the manipulation problem (Figure 7B; cf. Figure 5C). These torque values rapidly brought the MAE of the movement toward 0 (Figure 7C). When the synaptic weights were stabilized, the PC and DCN exploited their whole firing frequency range (Figure 7D). Thus, MF-DCN and PC-DCN plasticity allowed the system to efficiently self-rescale for optimal performance. Movies of learning simulations during manipulation of a 10-kg load are shown in the Supplemental Material.

### DCN synaptic plasticity improves predictive mass manipulation

To further evaluate the effectiveness of the DCN learning rules, we considered how the difference between the *predicted* and *actual* manipulated mass influenced the accuracy of movement. To this end, learning trials with different payloads (0.5, 1.5, 2.5, 6, or 10 kg) were performed testing four different cerebellar model configurations. This made it possible to test the impact of adaptation occurring at multiple synaptic sites: (i) plasticity only at PF-PC synapses, (ii) plasticity at PF-PC and MF-DCN synapses, (iii) plasticity at PF-PC and PC-DCN synapses, and (iv) plasticity at PF-PC, MF-DCN, and PC-DCN synapses.

The synaptic weights that were not allowed to change were set at their theoretical values pre-calculated for the accurate manipulation of 10-kg masses. In this way both MFs and PCs were able to provide enough excitation and inhibition, respectively, in order to avoid saturation at DCN. These experiments allowed us to evaluate the complementary and cooperative role of the different plasticities.

**Figure 8:**
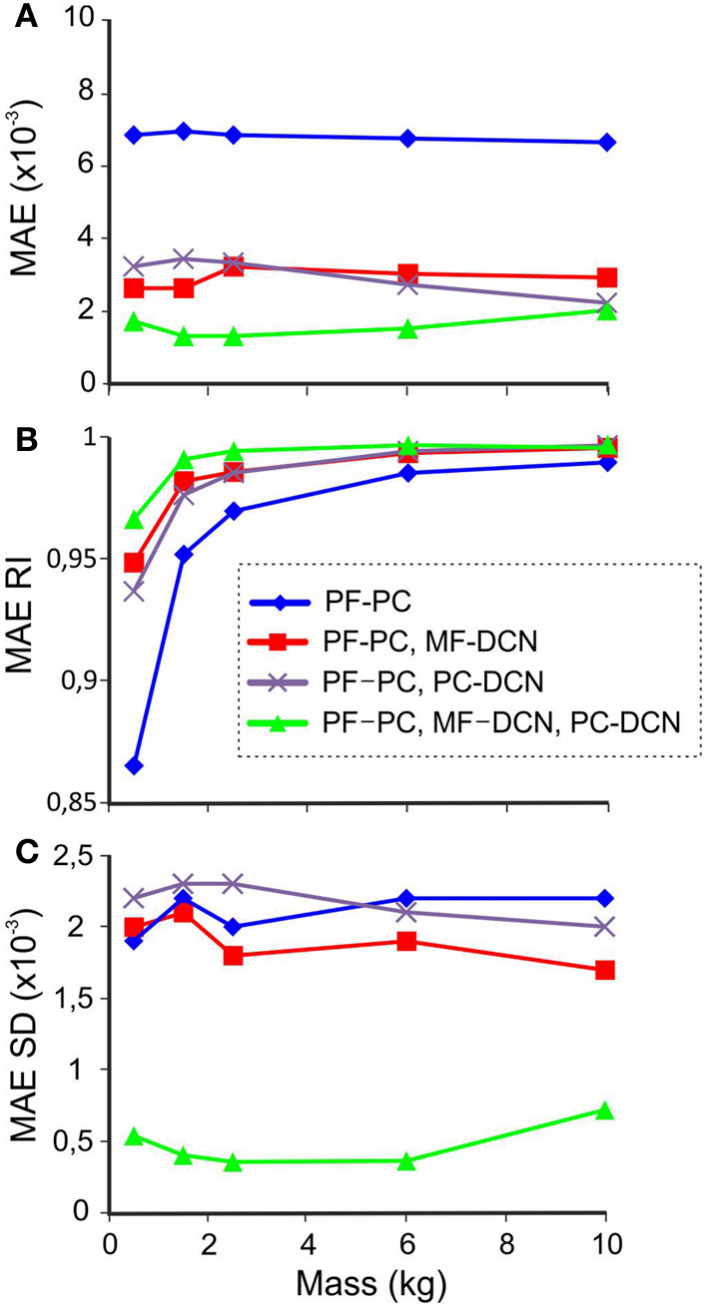
**Performance with different masses.** In synaptic connections with fixed weights, the values correspond to the 10-kg set up to avoid saturation during manipulation of heavier masses. The last 100 of 1500 trials during learning processes were used for MAE estimation with different combinations of active learning rules at different masses. **(A)** MAE **(B)** MAE_*RI*_ (1 is perfect error correction by the cerebellar action and 0 is no correction), **(C)** MAE standard deviation (*SD*).

For each combination of plasticities and masses, the learning process was simulated during 1500 trials, and the MAE at each joint was calculated at the end of the adaptation process. Figure 8A shows the average MAE during the last 100 trials. Plasticity at either MF-DCN or PC-DCN synapses reduced the average MAE, especially during the manipulation of lighter masses. Remarkably, enabling adaptation at just one of the two DCN afferent synapses was enough to improve manipulation precision. In line with this, plasticity at both MF-DCN and PC-DCN synapses simultaneously further increased the precision of manipulation. In order to obtain an objective evaluation of task performance independently of the manipulated mass, the “MAE reduction index” (MAE_*RI*_) was defined:

(9) $${\text{MAE}}_{RI}=1-\frac{{\text{MAE}}_{C+}}{{\text{MAE}}_{C-}}$$

where MAE_*C*+_ is the MAE of the manipulation task when using the cerebellar model corrective action and MAE_*C*−_ is the MAE in the absence of cerebellar adaptation (1 is the perfect error correction by the cerebellar action and 0 represents lack of correction). Using MAE_*RI*_ it is possible to compare the adjustment capacity of the cerebellar model independently of the payload.

The effect of the different cerebellar models during the manipulation of different masses is shown in Figure 8B. In all the cerebellar models, the trajectory error decreased when manipulating heavier masses. However, only the models incorporating both MF-DCN and PC-DCN plasticity were able to improve lighter mass manipulation. These results could be explained by evaluating the variability of MAE (Figure 8C). On incorporating plasticity at all the synapses (PF-PC, MF-DCN, PC-DCN), the variability of MAE after learning was markedly reduced, thus enhancing the stability of movements.

Thus, the model, by adjusting the MF-DCN and PC-DCN synaptic weights, thereby causing the indirect adjustment of PC activity to its widest possible firing range, improved the smoothness of the robot arm trajectory during the manipulation of objects with different masses. This made it possible to produce an accurate and stable learning process irrespective of the manipulated payload, thus providing the cerebellar system with the capability to self-adapt in order to manipulate different objects.

### Implicit representation of a double learning time scale

**Figure 9:**
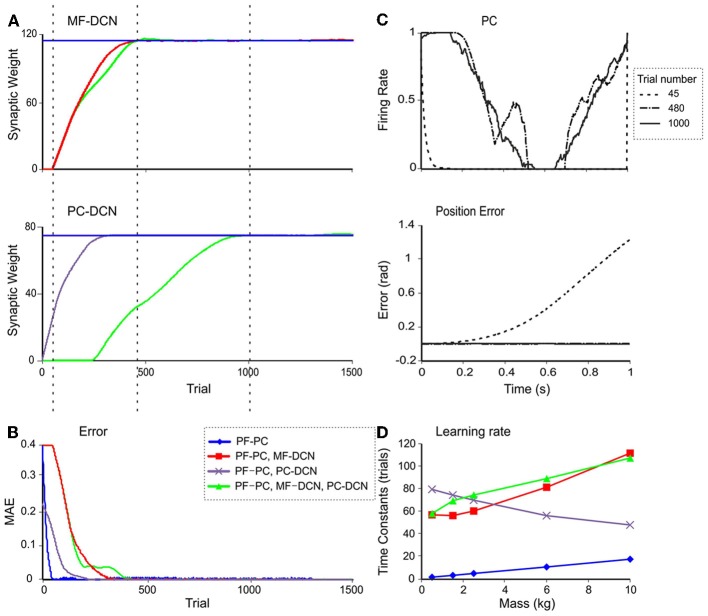
**Double time-scale learning. (A)** Evolution of MF-DCN synaptic weight *(top)*, and PC-DCN synaptic weight *(bottom)* during the learning process when a 10-kg payload is being manipulated. **(B)** Evolution of MAE in the same trials as in A. **(C)** PC firing rate *(top)* and actual position error *(bottom)* in joint 2 at three different times: after 45 trials *(gray line)*, 480 trials *(yellow line)*, and 1000 trials *(green line)*. **(D)** Time constants of the fitted exponential functions of the MAE with different learning rule settings and masses. The MAE evolution during the learning process has been fitted to exponential decaying functions. The time constants are shown when enabling the use of plasticity only at the PF-PC synapses (blue line), at the PF-PC and MF-DCN synapses (red line), at the PF-PC and PC-DCN synapses (purple line), and at the PF-PC, MF-DCN, and PC-DCN synapses (green line).

In order to verify whether the model supported the emergence of cerebellar learning consolidation, as indicated in recent behavioral and computational studies (Medina and Mauk, 1999; Ohyama et al., 2006; Xu-Wilson et al., 2009), the evolution of weight changes at DCN synapses was analyzed. During a 10-kg manipulation task (Figure 9A) the learning process was remarkably faster when DCN synaptic weights were pre-calculated. In this case, only the PF-PC synaptic weights, which stored the temporally correlated information, underwent adaptation, and learning was completed in around 50 trials. Otherwise, when weight changes at DCN synapses were enabled, learning required 200 trials (PC-DCN), 400 trials (MF-DCN), or 450 trials (PC-DCN and MF-DCN). In parallel, the MAE was remarkably reduced (Figure 9B).

Inspection of learning curves clearly showed that the learning process consisted of three different stages (Figure 9):

- The cerebellar model tried first to correct the initial error by using only PF-PC plasticity. This process took about 50 trials. When the MF-DCN or PC-DCN synaptic weights were not properly preconfigured, the PC activity saturated (Figure 9C).
- When PC activity did not completely remove the error, the MF-DCN synaptic weights were slowly adjusted after the PF-PC synaptic weights became saturated. This process started after 50 trials and took about 480 trials to complete. After stabilization of MF-DCN synaptic weights, the error was highly reduced; nonetheless, object manipulation remained imprecise.
- After about 300 trials, where the PC activity reached its maximum and in parallel with the MF-DCN weight evolution, the PC-DCN weights started increasing until the 1000th trial. Between 300 and 1000 trials the PC activity profile maintained a smooth shape and its trajectory remained close to the desired one.

Therefore, the model supported the existence of two different learning time scales consisting of: (i) a fast learning process, in which temporal information was inferred and stored at PF-PC synapses, and (ii) a slow learning process, in which the cerebellar excitatory and inhibitory gain values were adapted in the DCN and the manipulation precision increased. This second process was necessary only when the tool had never been manipulated before. During this process the MF-DCN and PC-DCN weights were simultaneously adapted at the same time as the PF-PC weights.

The fast and slow learning curves were fit to exponential decaying functions with time constants of 1–20 trials and 40–120 trials, depending on the object under manipulation (Figure 9D). The slow learning process could be further split into two components related to the MF-DCN and PF-PC connection with time-constants of 55–120 trials and 50–80 trials, respectively.

## Discussion

In this work, a theoretical model of the cerebellum is presented in the framework of a manipulation task, in which objects with different masses are moved along a desired trajectory. The main observation is that plastic mechanisms at DCN synapses effectively complement the learning capabilities of PF-PC synapses and contribute to the acquisition of the dynamics model of the arm/object plant. A proper synaptic weight adjustment at DCN synapses acts as a gain adaptation mechanism allowing the PFs to work within their most effective operative range, thus making the plasticity mechanisms between PFs and PCs more precise. This model, by incorporating distributed synaptic plasticity and by generating closed-loop simulations, allowed progressive error reduction based on feedback from the actual movement and accounted for three main theoretical aspects of cerebellar functioning.

First, the results support the principle that the cerebellum operates as a corrective inverse dynamic model (Schweighofer et al., 1996a,b, 1998b; Spoelstra et al., 2000). In the present model, the cerebellar granular layer was effectively implemented as a non-recurrent state generator (Yamazaki and Tanaka, 2007), in which the states correspond to the offset from stimulus onset implementing a *labeled-line coding* scheme. The granular layer states are then correlated with the error-based teaching signal received through the CFs. Thus, the model can be considered a particular case of an adaptive filter (Dean et al., 2009), in which the base functions in the granular layer are Dirac-deltas (impulse functions) with different delays for each granular cell or, in other words, a set of granular cells responding to different input stimuli along an arm trajectory trial (cf. D'Angelo and De Zeeuw, 2009).

Secondly, in the model, PF-PC plasticity temporally correlates the input state (or its representation in PFs) and the error estimation obtained during execution of the manipulation task. Instead, MF-DCN and PC-DCN plasticities store the excitatory and inhibitory gain of the neural network required to generate accurate correction of movement. Thus, the DCN afferent synapses infer the main properties of the object under manipulation, while the PF-PC synapses store the temporal characteristics of the task. As a consequence of this, plasticity at DCN synapses provides a homeostatic mechanism capable of keeping PC activity at its optimal range during learning. This effect can be observed in closed-loop simulations allowing progressive error reduction based on feedback from the actual movement.

Thirdly, the model supports the existence of a learning consolidation process, which has been demonstrated in behavioral experiments in human saccades (Brashers-Krug et al., 1996; Shadmehr and Brashers-Krug, 1997; Shadmehr and Holcomb, 1997; Xu-Wilson et al., 2009). While the cerebellar cortex plays a fundamental role at initial learning stages, the consolidation process seems to occur elsewhere. Our model provides a possible explanation of the learning consolidation process, locating it in the cerebellar nuclei. In our model, PF-PC plasticity evolves rapidly, while DCN plasticity evolves more slowly, because it depends on the previous evolution of plasticity at the PF-PC synapse itself. Therefore, our model naturally implements a double time-constant plasticity mechanism.

### The impact of plasticity at DCN synapses on adaptable gain control

Several experimental studies have reported LTD and LTP in DCN neurons (Morishita and Sastry, 1996; Aizenman et al., 1998; Ouardouz and Sastry, 2000; Bagnall and du Lac, 2006; Pugh and Raman, 2006) and a few hypotheses have been advanced about the role they play in the whole network. In previous studies, (Medina and Mauk, 1999, 2000) it was suggested that MF-DCN plasticity provides a mechanism for consolidating time-correlated information in the cerebellum and proposed that PC activity could drive the DCN learning process. Our model extends this hypothesis to the process of gain consolidation. Moreover, our model includes the possibility, by using a PC-driven learning rule, of storing gain information at PC-DCN connections. A model proposed for the VOR suggested that combined plasticity at the MF-DCN and PC-DCN synapses plays an important role in learning consolidation (Masuda and Amari, 2008). Our model further suggests that simultaneous MF-DCN and PC-DCN plasticity enhances movement precision in a manipulation task using a simulated robotic arm.

On the mechanistic level, our experimental approach allows different roles to be attributed to the different plasticity sites: PF-PC plasticity could act as a *time correlator* between the actual input state and the system error, while MF-DCN and PC-DCN plasticity together generated the *gain controller*. It is also possible that MF-DCN plasticity operates, at least in part, as a *state correlator*, as suggested previously (Masuda and Amari, 2008). Therefore, for improved performance, different aspects of computation have to be distributed over multiple adaptable network nodes.

### Biological realism and model limits

Before considering the further implications of this cerebellar model, its plausibility needs to be examined, analyzing the system design, learning rules, and coding strategies.

1. In this model we implemented the PC as a table correlating granular layer states with output torques evolving through the learning process. This, in conjunction with the PF-PC learning rule, allows the PC to behave as a state-error correlator. However, PC recordings in awake animals (Lisberger and Fuchs, 1978; Van Kan et al., 1993; Escudero et al., 1996; Cheron et al., 1997; Medina and Lisberger, 2009) suggest that PCs are more complex than state-error correlators. In the present model, given the high level of abstraction, it is impossible to evaluate PC features in terms of spike patterns. Inferences about signal coding in PCs would probably require the incorporation of realistic cerebellar network models into the system controller.
2. Since the learning rules used here at the MF-DCN and PC-DCN synapses depend only on PC and DCN activity, our model of gain control is compatible with different approaches to the distal error problem. Following the detailed descriptions provided on potential error detection mechanisms in the IO (Ito, 2013), the IO was assumed to receive both desired state information (encoding desired joint positions and velocities) conveyed by the motor cortex (Saint-Cyr, 1983) and actual state information (encoding actual joint positions and velocities) conveyed by the afferent sensory pathways, e.g., by the external cuneate nucleus concerning tactile and proprioceptive signals (Berkley and Hand, 1978; Molinari et al., 1996). This choice was supported by a computational model of the IO, which showed that the IO can indeed compare incoming signals (De Gruijl et al., 2012). It should be noted that alternative solutions to the distal error problem can be envisaged (Jordan and Rumelhart, 1992; Kawato, 1999), provided that PC activity saturates when the MF-DCN and PC-DCN weights are not properly tuned.
3. We used cerebellar feedback to correct the actual movement and we assumed that the teaching signal comes only through the CFs. However, there are indications that cerebellar feedback is also reverberated to the motor cortex (Kawato et al., 1987; Siciliano and Khatib, 2008), and some investigations suggest that the teaching signal is also received and correlated at the granular layer level (Krichmar et al., 1997; Kistler and Leo van Hemmen, 1999; Anastasio, 2001; Rothganger and Anastasio, 2009). The introduction of these elements is expected to increase the level of flexibility in motor control and learning.
4. We did not include the basal ganglia in our system controller. Recent evidence has suggested the existence of di-synaptic pathways connecting the cerebellum with the basal ganglia (Bostan et al., 2013). Both cerebellum (Swain et al., 2011) and basal ganglia (Bellebaum et al., 2008) have been suggested to contribute to reward-related learning tasks, but how these subsystems interact and reciprocally improve their operations remains an open issue.
5. We assumed that PF-PC plasticity tends to saturate toward LTP and that salient codes are stored when the CFs drive plasticity toward LTD at specific synapses. This mechanism could correspond to classical postsynaptic LTD (Márquez-Ruiz and Cheron, 2012) coupled with presynaptic LTP (Gao et al., 2012). The effectiveness of this core plasticity mechanism could be extended through multiple forms of LTP and LTD occurring at the PF-PC synapses and could be integrated with the inhibitory role played by MLIs (Wulff et al., 2009). MF-DCN and PC-DCN plasticity is implemented according to principles set out elsewhere (Medina and Mauk, 2000; Masuda and Amari, 2008). In our model, MF-DCN LTD followed increased PC activity. The full mechanism would comprise a secondary DCN spike increase through a rebound mechanism (Pugh and Raman, 2006), but this was irrelevant at our spike-less modeling level. Similarly, other details about the mechanisms of plasticity have not been applied. It remains to be established whether a biologically precise representation of plasticity mechanisms (e.g., Solinas et al., 2010) might modify the core conclusion of this model.
6. LTP and LTD between MFs and GCs have been shown to occur in slice experiments (D'Angelo et al., 1999; Armano et al., 2000; Maffei et al., 2002; Rossi et al., 2002; Sola et al., 2004; Gall et al., 2005; Mapelli and D'Angelo, 2007) and in vivo (Roggeri et al., 2008). However, the inclusion of granular layer LTP and LTD (Hansel et al., 2001) in a biologically realistic scenario would require (i) definition of the learning rules and teaching signals through the MFs (e.g., see D'Errico et al., 2009), (ii) definition of the spatiotemporal organization of the granular layer activity (D'Angelo, 2011; D'Angelo and Casali, 2012; D'Angelo et al., 2013; Garrido et al., 2013), and (iii) introduction of an explicit representation of spike timing (Nieus et al., 2006; D'Angelo and De Zeeuw, 2009). It has been suggested that MF-GC LTP and LTD, in conjunction with GC intrinsic plasticity and regulation of GoC–GC synaptic weights, could improve the learning capabilities of the system in target-reaching tasks (Schweighofer et al., 2001). In general, this hypothesis on the granular layer is compatible with the present model. Indeed, the labeled-line coding scheme that our model implements in the granular layer (Figure 2) can be seen as a particular case of sparse coding (although it is not very efficient in terms of the number of cells required to represent multiple states). Recent discoveries have revealed that sparse coding in the granular layer is related to the amount of GCs available for a particular task (Galliano et al., 2013). Our model, in fact, represents an extreme case of this hypothesis in which the population of GCs is so extensive that each PF encoded a unique non-recurrent condition. Moreover, it has been shown that the same GC can receive convergent inputs from proprioceptive sensory pathways coming from the external cuneate nucleus and efferent motor copies coming from the cerebral cortex via the pontine nucleus (Huang et al., 2013). In previous studies we already predicted that multi-modal information in the GCs could improve state representation capabilities (and, as a consequence, manipulation performance) in a non-adaptive model of the granular layer (Luque et al., 2011a). The development of a cerebellar model accounting for all these discoveries in the granular layer would require the use of realistic models implementing synaptic plasticity mechanisms and managing spike information (Solinas et al., 2010; Luque et al., 2011b,c). The integration of the present model into a spike-timing computational scheme including MF-GC plasticity rule remains a future challenge.

### Theoretical implications

This model has been conceived in order to be simple enough to become mathematically tractable while, at the same time, including salient properties of the system so as to retain its links with biology. In this sense it lies halfway between a classical black-box model and a realistic biological model. A non-trivial consequence of the way the model is constructed is that of providing a theoretical explanation for DCN plasticity, which increases cerebellar adaptable solutions. Moreover, this model could be compared to prototypical cases elaborated for dynamic neural networks (Spitzer, 2000; Hoellinger et al., 2013). In these networks, learning of complex tasks is better accomplished when the number of hidden neurons increases, as they form complex categories that are needed to interpret the multi-parametric input space. In the cerebellar network, the hidden units could intervene at different levels, including that of GCs lying between extracerebellar neurons and PCs, PCs lying between GCs and DCN, and also GoCs or MLIs in their respective subcircuits. In fact, extrapolation from theoretical works is limited by several biological constraints. For example, category formation is probably much more efficient in PCs than in GCs given the 10^5^ higher number of inputs in the PCs than in GCs, however there are many more GCs than PCs, and this results in a delicate balance between these cell types (the issue dates back to the seminal work of Marr, 1969). Conversely, GoCs and MLIs could implement exclusive-or (XOR) hidden layers, as suggested by experimental network analysis (Mapelli et al., 2010; Solinas et al., 2010). Moreover, PCs make synaptic connections with adjacent PCs through axonal collaterals suggesting that self-organizing properties might emerge in the molecular layer.

It should be noted that theoretical networks are oversimplified compared to the cerebellar model presented herein. For example, in Hoellinger's network plasticity can change the synapse from excitatory to inhibitory, connections are all-to-all, and gain and timing are stored in the same synapse (Hoellinger et al., 2013). A complementary step will be the inclusion of spiking dynamics, through the use of realistic network models (D'Angelo et al., 2009; Garrido et al., 2013). In this way, the implications of physiology (i.e., the role of the inhibitory PC collaterals, the complex structure of the PC dendritic tree and the operation of DCN cells with their characteristic postsynaptic rebounds) will be fully addressed.

## Conclusions

This model proposes a plausible explanation on how multiple plasticity sites, including the PF-PC and the MF-DCN and PC-DCN synapses, may effectively implement cerebellar gain control. According to the proposed model, distributed synaptic plasticity implements a gain controller, which (i) is *self-adaptable*, i.e., automatically rescales as a function of the manipulated masses over a large dynamic range, (ii) operates over *multiple time scales*, i.e., accounts for fast learning of time correlations and for subsequent gain consolidation, and (iii) improves learning accuracy and precision. These functions can be partly separated: the PF-PC synapse is suggested to operate mostly as a time correlator, while gain is more effectively regulated in DCN afferent synapses under PC control. In this way, time correlation and gain can be partially processed and stored independently. This organization of learning could explain the impact of genetic mutations impairing plasticity at cerebellar synapses. Indeed, irrespective of the specific synaptic plasticity mechanism involved (be it in the granular layer, molecular layer or DCN), transgenic mice bearing LTP or LTD alterations show deficits in cerebellar-related behavior and learning. However, the learning of timing and gain appear to be differentially affected, revealing that processing of these two components of learning are at least partially segregated (for a review see Boyden et al., 2004; Gao et al., 2012). Finally, it should be noted that the coexistence of fast and slow learning mechanisms can be reconciled with the double time-scale phenomenological model of learning proposed by Shadmehr and Mussa-Ivaldi (2012), which has been proposed to depend on localization of a fast learning process in the PF-PC synapse and a slower one in the DCN afferent synapses (Medina and Mauk, 1999; Medina et al., 2000).

A controller with distributed plasticity is convenient from a system designer's point of view, since it allows efficient adjustment of the corrective signal regardless of the dynamic features of the manipulated object and of the way it affects the dynamics of the arm plant involved. It should be noted that the adaptation mechanism adopted herein is not constrained to any specific plant or testing framework, and could therefore be extrapolated to other common testing paradigms like EBCC and the VOR. In order to do so, further details may be added to the model accounting for specific synaptic plasticity mechanisms and circuits involved in the different learning processes.

### Conflict of interest statement

The authors declare that the research was conducted in the absence of any commercial or financial relationships that could be construed as a potential conflict of interest.

## Supplementary material

Movie S1 **Learning simulation, joint-1-related activity and weight evolution during manipulation of a 10-kg load.** Simulations were carried out using all the plasticity mechanisms (PF-PC, MF-DCN, and PC-DCN) along 1000 trials. Only 1 every 10 trials is shown. The movement has been recorded in real-time (each trial lasts 1 s) evidencing the difficulty of the task. *(top left)* 3D view of the actual *(black)* and desired *(red)* robot end-effector trajectory in Cartesian coordinates. *(medium left)* Ideal *(dotted lines)* and actual *(solid lines)* corrective torques during the current trial for joint 1 *(blue)*, 2 *(red)*, and 3 *(green)*. *(bottom left)* Evolution of the MAE. *(top right)* Evolution of four randomly chosen PF-PC synaptic weights. *(second to fifth rows right)* Evolution of PC activity, DCN activity, MF-DCN, and PC-DCN synaptic weights related to joint 1 *agonist (solid line)* and antagonist *(dotted line)* muscles. Note that, at the end of learning, joint-1 DCN neurons provided higher corrective torques to the antagonist muscle during the first half of the trial and to the agonist muscle during the second half of the trial.

Movie S2 **Learning simulation, joint-2-related activity and weight evolution during manipulation of a 10-kg load.** Simulations were carried out using all the plasticity mechanisms (PF-PC, MF-DCN, and PC-DCN) along 1000 trials. Only 1 every 10 trials is shown. The movement has been recorded in real-time (each trial lasts 1 s) evidencing the difficulty of the task. *(top left)* 3D view of the actual *(black)* and desired *(red)* robot end-effector trajectory in Cartesian coordinates. *(medium left)* Ideal *(dotted lines)* and actual *(solid lines)* corrective torques during the current trial for joint 1 *(blue)*, 2 *(red)*, and 3 *(green)*. *(bottom left)* Evolution of the MAE. *(top right)* Evolution of four randomly chosen PF-PC synaptic weights. *(second to fifth rows right)* Evolution of PC activity, DCN activity, MF-DCN, and PC-DCN synaptic weights related to joint 2 *agonist (solid line)* and antagonist *(dotted line)* muscles. Differently from what observed for joint 1, at the end of learning, joint-2 DCN neurons provided higher corrective torques to the agonist muscle during the whole trial.

Movie S3 **Learning simulation, joint-3-related activity and weight evolution during manipulation of a 10-kg load.** Simulations were carried out using all the plasticity mechanisms (PF-PC, MF-DCN, and PC-DCN) along 1000 trials. Only 1 every 10 trials is shown. The movement has been recorded in real-time (each trial lasts 1 s) evidencing the difficulty of the task. *(top left)* 3D view of the actual *(black)* and desired *(red)* robot end-effector trajectory in Cartesian coordinates. *(medium left)* Ideal *(dotted lines)* and actual *(solid lines)* corrective torques during the current trial for joint 1 *(blue)*, 2 *(red)*, and 3 *(green)*. *(bottom left)* Evolution of the MAE. *(top right)* Evolution of four randomly chosen PF-PC synaptic weights. *(second to fifth rows right)* Evolution of PC activity, DCN activity, MF-DCN and PC-DCN synaptic weights related to joint 3 *agonist (solid line)* and antagonist *(dotted line)* muscles. Similarly to what observed for joint 2, joint 3 corrective torques provided by DCN neurons were dominated by agonist muscle activity, but different gain values were provided with respect to joint 2.

## Appendix A. Ideal torque values and final synaptic weights

**Table A1:** **Theoretical torque values when manipulating different masses**.

**External masses**	**Torque (Nm) — Joint 1**		**Torque (Nm) — Joint 2**		**Torque (Nm) — Joint 3**	
	**Minimum**	**Maximum**	**Minimum**	**Maximum**	**Minimum**	**Maximum**
0.5 kg	−1.40	1.45	1.8	5.85	0.72	3.05
1.5 kg	−3.95	4.07	5.75	17.16	2.51	8.7
2.5 kg	−6.7	6.9	9.55	28.64	4.15	14.56
6 kg	−16	16.5	23.15	68.5	10.15	34.76
10 kg	−26.66	27.46	38.68	114.08	17	57.85

*The solution of the present manipulation problem required a continuous sinusoidal torque with different phases and amplitudes per each joint. The table shows the maximum and minimum corrective torques for each combination of joints and masses. Note that joint 1 includes both positive values (clockwise forces) and negative values (anti-clockwise) which should be applied by activating the pairs of agonist and antagonist muscles. Joints 2 and 3 required only the application of positive torques. Thus, most of the torques will be applied by the agonist muscles, requiring the antagonist muscles only for stabilization*.

**Table A2:** **MF-DCN synaptic weights**.

**External masses**	**Weight — Joint 1**		**Weight — Joint 2**		**Weight — Joint 3**	
	**Agonist**	**Antagonist**	**Agonist**	**Antagonist**	**Agonist**	**Antagonist**
0.5 kg	1.7	1.5	6	0	3.6	0
1.5 kg	4.2	4.1	17.4	0	9.3	0
2.5 kg	6.95	6.8	28.9	0	15.1	0
6 kg	16.3	16.9	68.9	0	35	0
10 kg	26.8	26.4	113.8	0	57.4	0

*The weights were obtained after a 10000-trial simulation involving manipulation of different masses with all learning rules enabled. After 10000 trials, the MF-DCN synaptic weights remained stable. Synaptic weights are represented for each muscle in the agonist/antagonist pairs. In joint 1, both clockwise and anti-clockwise corrective torques needed to be applied, so that agonist weights fitted the maximum torque and antagonist weights fitted the minimum torque values (ignoring the direction). In joints 2 and 3, the antagonist muscles were automatically disabled because only positive torques were required to achieve the desired correction (see Table A1)*.

**Table A3:** **PC-DCN synaptic weights**.

**External masses**	**Weight — Joint 1**		**Weight — Joint 2**		**Weight — Joint 3**	
	**Agonist**	**Antagonist**	**Agonist**	**Antagonist**	**Agonist**	**Antagonist**
0.5 kg	1.7	1.5	4.5	0	3.6	0
1.5 kg	4.2	4.1	11.9	0^*^	9.3	0^*^
2.5 kg	6.95	6.8	19.4	0^*^	11.71	0^*^
6 kg	16.3	16.1	45.8	0^*^	25.2	0^*^
10 kg	26.8	26.4	74.9	0^*^	40.1	0^*^

The weights were obtained after a 10000-trial simulation involving manipulation of different masses with all learning rules enabled. After 10000 trials, some of the PC-DCN synaptic weights were stabilized. However, the weights marked with (

* *) slowly decreased and reached their convergence values after the 10000 trials (up to 30000 trials depending on the mass). Synaptic weights are represented for each muscle in the agonist/antagonist pairs. In joint 1, both clockwise and anti-clockwise corrective torques needed to be applied, so that inhibition coming from the PC-DCN connection completely inhibited MF-DCN activity and synaptic weight values became similar to those of the MF-DCN synapse (cf.Table A2). In joints 2 and 3, the antagonist muscle inhibition was automatically disabled because no excitation was needed (cf. Table A2)*.

## Appendix B. Robotic arm description

The inverse dynamic equation defining the lightweight robot (LWR) is given by the expression:

(B1) $$\tau =M(Q)\cdot \ddot{Q}+C(Q)\cdot \left[\dot{Q}\dot{Q}\right]-D(Q)\cdot \left[{\dot{Q}}^{2}\right]+G(Q)+F(Q,\dot{Q})$$

where τ is the torque value vector to be applied by the robot joints, *Q*, $\dot{Q}$, and $\ddot{Q}$ are vectors representing the positions, velocities, and accelerations of the joints, $\left[\dot{Q}\dot{Q}\right]$ and [*Q*^2^] being vectors defined as follows:

(B2) $$\begin{array}{l} \left[\dot{Q}\dot{Q}\right]=[{\dot{Q}}_{1}\cdot {\dot{Q}}_{2},\ldots ,{\dot{Q}}_{1}\cdot {\dot{Q}}_{n},{\dot{Q}}_{2}\cdot {\dot{Q}}_{3},\ldots ,{\dot{Q}}_{2}\cdot {\dot{Q}}_{n},\ldots ,\\ \;{{\dot{Q}}_{n\;-\;1}\cdot {\dot{Q}}_{n}]}^{T} \end{array}$$

(B3) $$\left[{\dot{Q}}^{2}\right]={\left[{\dot{Q}}_{1}^{2},{\dot{Q}}_{2}^{2},\ldots ,{\dot{Q}}_{n}^{2}\right]}^{T}$$

where *n* represents the number of links included in the robotic arm, *M*(*Q*) represents the inertia matrix (the mass matrix), *C*(*Q*) is the Coriolis matrix, *D*(*Q*) represents the matrix of centrifugal coefficients, *G*(*Q*) is the gravity action on the joints and finally *F*(*Q*, $\dot{Q}$) represents the friction term. The friction term is crucial in controlling LWR arms with high-ratio gear boxes since conventional methodologies fail to control these robots without a massive modeling (van der Smagt, 2000). At the same time, the friction term can be differentiated in two terms; dry and viscous friction components obtaining:

(B4) $$F(Q,\dot{Q})=F_{D}(Q,\dot{Q})\pm F_{V}(Q,\dot{Q})$$

where *F_D_*(*Q*, $\dot{Q}$) and *F_V_*(*Q*, $\dot{Q}$) are the dry/viscous friction matrices, respectively. The first four terms of Equation B1 mainly include the inherent robot dynamic parameters (inertia matrix, Coriolis/centrifugal matrix, and gravitational force vector). These parameters are up to eleven per joint (inertia matrix is symmetrical):

1. Inertia tensor terms: (*xx*_*j*_, *xy*_*j*_, *xz*_*j*_, *yy*_*j*_, *yz*_*j*_, *zz*_*j*_)
2. Center of Mass: (*mx*_*j*_, *my*_*j*_, *mz*_*j*_)
3. Mass: (*M*_*j*_)
4. Motor Inertia: (*I*_*j*_)

where *j* ranges from 1 to the number of joints (3 in our model). These parameters are usually grouped according to these four categories in order to make the computational task easier (Khalil and Dombre, 2004). For our particular LWR (Albu-Schäffer et al., 2007), the nominal values obtained applying parametric methods (Bona and Curatella, 2005) are shown in Tables B1–B3.

**Table B1:** **Inertia tensor parameters (*kg* · *m*^2^)**.

**Joint**	***xx_j_***	***xy_j_***	***xz_j_***	***yy_j_***	***yz_j_***	***zz_j_***
1	0.0216417	0	0	0.0214810	0.0022034	0.0049639
2	0.0244442	0	0	0.0052508	0.0036944	0.0239951
3	0.0213026	0	0	0.0210353	0.0022204	0.0046970
4	0.0231668	0	0	0.0048331	0.0034937	0.0227509
5	0.0081391	0	0	0.0075015	0.0021299	0.0030151
6	0.0033636	0	0	0.0029876	0	0.0029705
7	0.0000793	0	0	0.0000783	0	0.0001203

**Table B2:** **Centers of masses (*m*), masses (*kg*) and motor inertias (*kg* · *m*^2^)**.

**Joint**	***mx_j_***	***my_j_***	***mz_j_***	***m_j_***	***I_j_***
1	0.0	0.01698	−0.05913	2.7082	415.50e-6
2	0.0	0.11090	0.01410	2.7100	415.50e-6
3	0.0	−0.01628	−0.06621	2.5374	361.60e-6
4	0.0	−0.10538	0.01525	2.5053	138.50e-6
5	0.0	0.01566	−0.12511	1.3028	54.10e-6
6	0.0	0.00283	−0.00228	1.5686	60.08e-6
7	0.0	0.0	0.06031	0.1943	60.08e-6

**Table B3:** **Friction parameters: dry friction (*N* · *m*) and viscous friction (*N* · *m* · *s/rad*)**.

**Joint**	***F_Dj_***	***F_Vj_***
1	∓0.35	2.0e-3
2	∓0.35	1.69800e-3
3	∓0.35	1.66000e-3
4	∓0.35	2.40000e-3
5	∓0.35	1.80000e-3
6	∓0.35	1.20000e-3
7	∓0.35	1.20000e-3

## Abbreviations

PF: parallel fiber
MF: mossy fiber
CF: climbing fiber
GC: granule cell
GoC: Golgi cell
PC: Purkinje cell
DCN: deep cerebellar nuclei
VN: vestibular nuclei
IO: inferior olive
MLI: molecular layer interneuron
EBCC: eye-blink classical conditioning
VOR: vestibule-occular reflex
MAE: mean average error.
